# A global taxonomy of interpretable AI: unifying the terminology for the technical and social sciences

**DOI:** 10.1007/s10462-022-10256-8

**Published:** 2022-09-06

**Authors:** Mara Graziani, Lidia Dutkiewicz, Davide Calvaresi, José Pereira Amorim, Katerina Yordanova, Mor Vered, Rahul Nair, Pedro Henriques Abreu, Tobias Blanke, Valeria Pulignano, John O. Prior, Lode Lauwaert, Wessel Reijers, Adrien Depeursinge, Vincent Andrearczyk, Henning Müller

**Affiliations:** 1 CISUC, Department of Informatics Engineering, University of Coimbra, Pólo II, Pinhal de Marrocos, Coimbra, 3030-790 Portugal; 2 IPO-Porto Research Centre, Rua Dr. António Bernardino de Almeida, Porto, 4200-072 Portugal; 3 University of Applied Sciences of Western Switzerland (HES-SO Valais), Rue du Technopole 3, Sierre, 3960 Valais Switzerland; 4 Department of Computer Science, University of Geneva (UniGe), Route de Drize 7, Carouge, 1227 Vaud Switzerland; 5 Department of Radiology and Medical Informatics, University of Geneva (UniGe), Rue Gabrielle-Perret-Gentil 4, Geneva, 1211 Vaud Switzerland; 6 Faculty of Social Science, Centre for Sociological Research, Parkstraat 45 bus, Leuven, 3000 Belgium; 7 Robert Schuman Centre, European University Institute, Via Boccaccio 121, Florence, 50133 Italy; 8 Institute of Logic, Language and Computation, University of Amsterdam, Spui 21, Amsterdam, 1012WX Netherlands; 9 Department of Nuclear Medicine and Molecular Imaging, Lausanne University Hospital, Rue du Bugnon 46, Lausanne, 1011 Vaud Switzerland; 10 IBM Research Europe, 3 Technology Campus, Dublin, D15 HN66 Ireland; 11 Centre for IT and IP Law, KU Leuven, Sint-Michielsstraat 6, Leuven, 3000 Belgium; 12 Department of Data Science and AI, Monash University, Wellington Rd, Clayton VIC, Melbourne, 3800 Australia; 13 Institute of Philosophy, KU Leuven, Kardinaal Mercierplein 2, bus 3200, Leuven, 3000 Belgium

**Keywords:** Interpretability, Explainable artificial intelligence, Machine learning

## Abstract

Since its emergence in the 1960s, Artificial Intelligence (AI) has grown to conquer many technology products and their fields of application. Machine learning, as a major part of the current AI solutions, can learn from the data and through experience to reach high performance on various tasks. This growing success of AI algorithms has led to a need for interpretability to understand opaque models such as deep neural networks. Various requirements have been raised from different domains, together with numerous tools to debug, justify outcomes, and establish the safety, fairness and reliability of the models. This variety of tasks has led to inconsistencies in the terminology with, for instance, terms such as *interpretable*, *explainable* and *transparent* being often used interchangeably in methodology papers. These words, however, convey different meanings and are “weighted" differently across domains, for example in the technical and social sciences. In this paper, we propose an overarching terminology of interpretability of AI systems that can be referred to by the technical developers as much as by the social sciences community to pursue clarity and efficiency in the definition of regulations for ethical and reliable AI development. We show how our taxonomy and definition of interpretable AI differ from the ones in previous research and how they apply with high versatility to several domains and use cases, proposing a—highly needed—standard for the communication among interdisciplinary areas of AI.

## Introduction

**Fig. 1:**
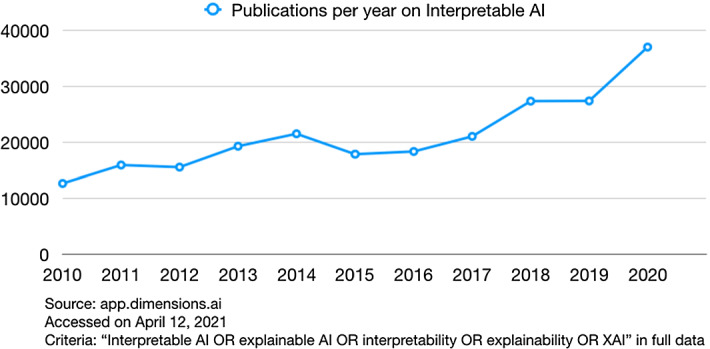
Trends of the publications containing “interpretable AI" or “explainable AI" as keywords

The last decade saw a sharp increase in research papers concerning interpretability for Artificial Intelligence (AI), also referred to as eXplainable AI (XAI). In 2020, the number of papers containing “interpretable AI", “explainable AI", “XAI", “explainability", or “interpretability" has increased to more than three times that of 2010, following the trend shown in Fig. 1.

**Fig. 2:**
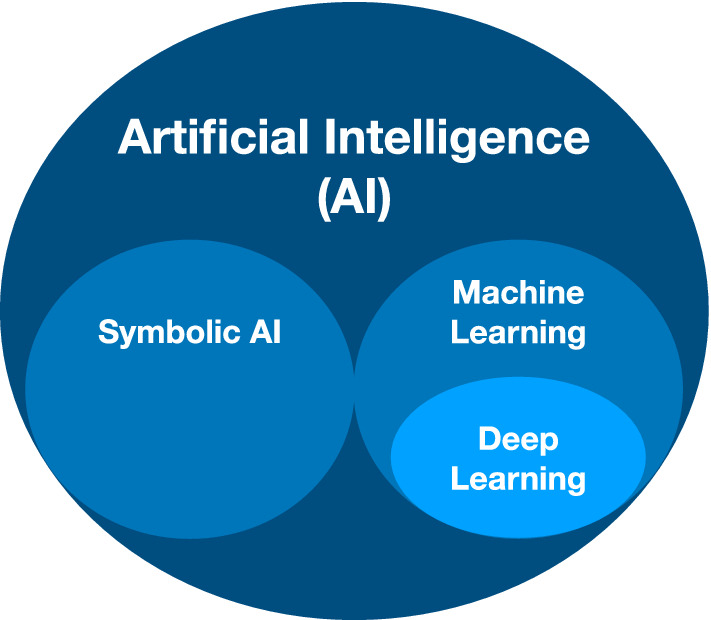
Graphical representation of Artificial Intelligence, Machine Learning, and Deep Learning adapted from https://www.intel.com

Being applied to an increasingly large number of applications and domains, AI solutions mostly divide into the two approaches illustrated in Fig. 2. On the one side, we have *Symbolic AI*, symbolic reasoning on knowledge bases as an important element of automated intelligent agents, which reflect the humans’ social constructs into the virtual world (Russell and Norvig 2002). To communicate intuitions and results, humans (henceforth agents) tend to construct and share rational explanations, which are means to match intuitive and analytical cognition (Omicini 2020). On the other side, Machine Learning (ML) and Deep Learning (DL) models reach high performance by learning from the data and through experience. The complexity of the tasks in both approaches has increased over time, together with the complexity of the models being used and their opacity. A rising interest in interpretability came with the increasing opacity of the systems and with the frequent adoption of "black-box" methods such as DL, as documented by multiple studies (Miller 2019; Lipton 2018; Tjoa and Guan 2020; Murdoch et al. 2019; Chromik and Schuessler 2020; Arrieta et al. 2020; Adadi and Berrada 2018; Rudin 2019; Arya et al. 2019; Mittelstadt et al. 2019).

A strong condition to ensure the reliable use of AI is improving the understanding of its internal mechanics, particularly when complex DL models are deployed. As the previous studies on interpretability point out, understanding the decision-making of an AI system is a non-trivial task that spans over three areas, namely understanding the task, the performance metric used by the model and the type of experience being used. With the intent of improving the interpretability within these three areas, a large number of requirements, tools and techniques have been developed in different application fields, leading to inconsistent use of the terminology. Interpretability is often confused with more abstract notions of fairness, privacy and transparency (Weller 2019). These terms do not have a clear and unique definition and the understanding of these terms may differ depending on the domain and context. Similarly, the words interpretable and explainable have been used interchangeably in some articles (Miller 2019; Lipton 2018), while others use a strong distinction between the two terms (Rudin 2019). Undoubtedly, there is a link between the act of interpreting and that of explaining, as shown by the etymology of the words themselves (that we report in Table 3). Interpretability has been presented as “explaining or presenting in understandable terms to a human", “providing explanations" to humans (Miller 2019) and “assigning meaning to an explanation" (Palacio et al. 2021). For (Rudin 2019), however, there is a strong distinction between *interpreting* and *explaining* since models may be developed to directly encompass the ability to explain their decision-making. In this case, interpretability refers to meeting the transparency requirement at the task definition level, whereas explanation refers to a post-hoc (after training) evaluation of the model understandability.

The different perspectives about the technical terminology are discussed in several papers within the specific context of explainable AI and ML design, finding difficult integration within the other domains that are driving and shaping AI development. Policies for funding and regulating AI research also refer to concepts such as *transparency*, *explicability*, *reliability*, *informed consent*, *accountability*, and *auditability* of the systems (Bibal et al. 2020, 2020; Edwards and Veale 2017). Clarifying what these terms refer to and unifying the social and technical perspectives on these aspects is fundamental to determine directions for progress and to encourage cross-disciplinary discussion and interaction on AI developments. Fields that analyzed the impact of technologies over the centuries such as cognitive sciences, sociology, philosophy and ethics constitute invaluable resources of knowledge from which it is possible to evaluate and understand how human trust evolves over time and how it can be built to motivate the adoption of new technologies. If the use of a global terminology is adopted by these disciplines, then a broader range of possibilities can open, encouraging the design of interpretability tools that are not only useful and understandable to ML developers but to a wider audience ranging from the final decision-maker to anyone affected by this decision (Tonekaboni et al. 2019).

The contributions of this paper are the following: (i) we collect the perspectives on the interpretable AI terminology from a large number of experts, reporting the results of the interdisciplinary collaboration with 8 disciplines in the social and technical sciences; (ii) we propose a taxonomy and interdisciplinary definitions for interpretability and interpretable AI that can be used in multiple contexts; (iii) we propose the study of a use case in the medical field to demonstrate the relevance of unifying perspectives and adopting a common terminology.

## Related work

Several papers in the literature proposed a taxonomy of interpretable AI. Table 1 reviews in chronological order the numerous definitions that were given in the ML literature for *interpretable*, *explainable*, *transparent*, *decomposable* and *intelligible*. While trying to be as complete as possible, we clarify that this table is not exhaustive. We excluded from this review the papers that defined the taxonomy for developing a single technique. Discordance can be noticed on the meaning assigned to the terms by the papers in this collection, with major dividing points emerging on the words: (i) interpretable and explainable; (ii) transparency and decomposability ; (iii) intelligible and interpretable;

The terms interpretable and explainable are equated, for example, by several researchers (Miller 2019; Adadi and Berrada 2018; Arya et al. 2019; Clinciu and Hastie 2019; Murdoch et al. 2019; Vered et al. 2020). An even broader number of papers describes a clear distinction between these two terms (Rudin 2019; Lipton 2018; Biran and Cotton 2017; Montavon et al. 2018; Mittelstadt et al. 2019; Chromik and Schuessler 2020; Arrieta et al. 2020; Palacio et al. 2021), suggesting that a distinction between these two terms is more popular among researchers. As for interpretability, multiple definitions exists also within the context of explainability, for which we refer the reader to the systematic review by Vilone and Longo (2020). The work by Arrieta et al. (2020), for instance, distinguishes interpretability from explainability, which is defined as a human-understandable interface that exists between the user and the system. Transparency is used in multiple papers with the meaning described by Lipton (2018) of model decomposability (Lipton 2018; Clinciu and Hastie 2019; Chromik and Schuessler 2020). In other papers, this term is used as a synonym for interpretability (Murdoch et al. 2019; Arrieta et al. 2020) or for functional understanding of the model (Mittelstadt et al. 2019). Rudin et al. (2019) define transparency as models with particular properties such as monotonicity since these models are transparent in the way their variables are jointly related. Finally, the concept of intelligible model equated to that of an inherently interpretable model in Arya et al. (2019), while it is used meaning the introduction of interpretability constraints in the model design in Clinciu and Hastie (2019); Montavon et al. (2018).

None of the papers in Table 1 considers the taxonomy used by policymakers, regulators, philosophers and sociologists discussing the impact of AI on society and on the research community. The perspectives in this paper are discussed by experts in AI development and familiarity with ML. As a consequence, different definitions are used in social sciences. This paper reviews the existing definitions and gathers the perspectives from a multidisciplinary pool of experts to provide a taxonomy that can be used in multiple domains in a unique way that adapts to both the social and the technical sciences.

**Table 1:** Multiple taxonomies-part 1

Interpretable	Explainable	Transparent	Intelligible	Refs.
The system operations can be understood by a human, either through introspection or through a produced explanation	To show the rationale behind each step in the decision. It is linked to justification and affects user acceptance and satisfaction	Not mentioned	Not mentioned, although they refer to introspective explanations	Biran and Cotton (2017)
Ability to explain or to present in understandable terms to a human	Not mentioned	Not mentioned	Not mentioned	Doshi-Velez and Kim (2017)
A non-monolithic concept reflecting several distinct ideas.	Solely intended as post-hoc interpretability. Post-hoc explanations can be verbal, and visual	Understanding the mechanism by which the model works. Related to simulatability and decomposability.	Understandable models are sometimes called transparent	Lipton (2018)
A mapping of an abstract concept into a domain that the human can make sense of	Collection of features [$\dots$] that have contributed to produce a given decision	Achievable by both interpreting and explaining ML outcomes	Post-hoc interpretability should be contrasted to incorporate interpretability into the structure of the model.	Montavon et al. (2018)
Used more frequently than “explainable” by the ML community, referring to a powerful tool for justifying AI-based decisions	Not mentioned	Not mentioned	Understandability is characterized by no means of understanding the internal model functioning. Understandable is different from intelligible	Adadi and Berrada (2018)
The level to which an agent gains and can make use of both the information embedded within explanations given by the system and the information provided by the system’s transparency level	The level to which a system can provide clarification for the cause of its decisions/outputs	The level to which a system provides information about its internal workings or structure and the data it has been trained with	Not mentioned.	Tomsett et al. (2018)
Equated with “explainability”, it defines the degree to which an observer can understand the cause of a decision"	Establishing an interaction between the explainer and the explainee (i.e. the subject on the receiving end of an explanation), that is contextual and selective, based on small subset of causes	Briefly mentioned as interlinked to trust	Not mentioned	Miller (2019)
Acknowledgment of multifaceted definitions from earlier studies	Answering “why" and “why not" questions to improve the user’s mental model of the system. In other cases, equated to interpretable	Providing explanations on how the system works, clearly describing model structure, equations, parameter values and assumptions	A system that is “clear enough to be understood". It is challenging to understand how an AI system should be defined in order to be “intelligible" since this would require the clarification of “complex computational processes to various types of users"	Clinciu and Hastie (2019)
Broadly defined, referring to the extraction of relevant knowledge (visualization, language, or equation) about domain relationships contained in the data.	Used as a synonym of interpreting	A feature engineering process to enhance the analysis of model interpretability	Not mentioned	Murdoch et al. (2019)

**Table 2:** Multiple taxonomies-part 2

Interpretable	Explainable	Transparent	Intelligible	Refs.
Used interchangeably with explainable	Post-hoc explanations involve an auxiliary method after a model is trained. Self-explaining models generate local explanations that may not be directly interpretable	Not mentioned	A “directly interpretable" model, namely intrinsically understandable by most consumers	Arya et al. (2019)
It is a domain-specific notion that does not allow a general-purpose definition. An interpretable ML model is constrained in model form so that it is either useful to someone, or obeys structural knowledge of the domain [...]	Possibly unreliable and misleading, explanations are not faithful to what the original model computes. Often, they do not make sense nor do they provide enough detail to understand what the black box is doing	Fully transparent models are allowed to understand their variables and the related correlations	Not mentioned.	Rudin (2019)
It refers to the degree of human comprehensibility of a given black-box model or decision	It refers to the numerous ways of exchanging information about a phenomenon (a model’s functionality or the rationale and criteria for a decision) with multiple stakeholders	A model is transparent if its functionality can be comprehended in its entirety by a person	Not mentioned	Mittelstadt et al. (2019)
It is a passive characteristic of a model referring to the level at which it makes sense for a human observer (also referred to as transparency)	Any action or procedure to clarify the internal model functions	As in Lipton, described by Simulability, Decomposability and Algorithmic Transparency	Not mentioned. Understandable is different from intelligible	Chromik and Schuessler (2020)
It encompasses multiple concepts and definitions. Generally, it is associated with models with inherently interpretable behavior	It is intended as the generation of post-hoc explanations for black-box models	It is intended as an explanation of how the system works	Not mentioned	Arrieta et al. (2020)
Assigning meaning to an explanation	Process of describing one or more facts, facilitating the understanding of said facts by a human consumer	Not mentioned	Not mentioned	Palacio et al. (2021)
Assigning a subjective meaning to a model, object, or variable that is possible to be interpreted by the explainee	The activity of producing more interpretable objects manipulating symbolic information	Providing a clear representation of the black-box dynamics	Concerning the explainee, it is intended a successful consumption of an explanation	Ciatto et al. (2020)

## Methods

A round table public meeting was held online on April 29th, 2021 on “A Global Taxonomy for Interpretable AI"^1^. Endorsed by the AI4Media project within the European Union’s Horizon 2020 for research and innovation plan, this event was organized to bring together researchers from multidisciplinary backgrounds to collaborate on a global definition of interpretability that may be used with high versatility in the documentation of social, cognitive, philosophical, ethical and legal concerns about AI. A total of 18 experts were invited to participate in the event. The selection of the experts was tailored to obtain the most representative consortium of the fields dealing with Interpretable AI at the moment. The final pool of experts involved in this work also depended on the experts’ interests and their availability but the selection was by no means at all made in such a way to steer the discussion in the direction of a pre-agreed consensus. The experts were both internal members of the AI4media project and external non-affiliated members. The external experts were invited so as to obtain a balanced perspective on the topic that went beyond the purpose of the project itself. For each of the discussed disciplines, at least one external expert was included in the discussion. The selection was done based on the previous publication records on interpretable AI and on the reported interest and availability to participate in the study. In addition, attention was given to the inclusiveness in terms of gender and ethnicity of the experts. The experts represent institutions from eight different countries (of which two are non-european) and span from academia to industry and healthcare professionals.

The workshop was organized in two sessions, consisting of a round table discussion and a panel session with a question and answer format. The first session consisted of seven short talks of 12 minutes followed by 3 minutes for questions. The second session involved a panel of five experts discussing questions from the audience concerning the role and implications of AI and transparency. The workshop was streamed on YouTube^2^ and spectators were able to interact with the audience through a live chat.

The round table resulted in a solid basis for the work reported in this paper and steered further discussion and proposed future research directions. We hope that this work may constitute a first solid step towards finding a global consensus on the taxonomy for interpretable AI for both the social and the technical sciences.

## Results

### Etymology and existing definitions

Table 3 analyzes the etymology of frequently used words in the context of interpretable AI. Looking at the historical formation and the original meaning of a word can shed light on its roots and history, deepening the understanding of its meaning and the context in which it should be used. The word clue, for example, gains meaning from its intrinsic referral to Greek mythology. It originates from the Germanic word clew that indicates a ball of thread or yarn. Theseus used a clue of thread to find the exit of the Labyrinth. When people say “give me a clue", they refer to some helpful information and not the ball of yarn itself. Understanding the etymology of the words in the AI interpretability terminology can help in a similar way to better understand the meaning of each term and why one word is more appropriate than another in specific contexts.

**Table 3:** Analysis of the etymology of the terms related to interpretability

ID	Word	Etymology		ML Definition
1	Interpretability, Interpretable	From late Latin interpretabilitis from Latin interprĕtor, interprĕtāri (to interpret)	To interpret, comment, explain, expose, illustrate, to translate	To translate, expose, and comment on the generation process of one or multiple ML systems outcomes, making the overall process understandable by a human
2	Explainability, Explainable	From 1600 use of explain + -able adapted from Latin explāno, explānāre	To explain, clarify, expose, illustrate, state clearly	To indicate with precision, to illustrate what features or high-level concepts were used by the ML system to generate predictions for one or multiple inputs. In intelligent agent systems: possibly iterative process of symbolic knowledge manipulation to make it interpretable
3	Transparency, Transparent	Medieval Latin adaptation of the words trans (on the other side) and pārĕo, pārēre (to appear, to show)	To see through	A *transparent* ML system has a non-opaque output-generation process where the role of the individual components, the learned paradigms, and the overall behavior of the model are known and can be simulated by a human user
4	Intelligibility, Intelligible	From Latin intellegibilis, intellegibilis, II class adjective	To understand, comprehend, decipher	An intelligible ML system is an understandable system with inherent interpretability
5	Accountability, Accountable.	From 1770 use of accountable + -ity, adapted from Old French acont derived from Latin compŭto, compŭtāre, which has multiple meanings including to count, to estimate, to judge and to believe.	Used from the 1610s with the sense of “rendering an account", meaning providing a statement answering for conduct.	An accountable ML system is expected to justify its outcomes and behavior
6	Reliability, Reliable	From Scottish of the 1560s “raliabill", derived from Old French relier a derivation of the Latin rĕlĭgo, rĕlĭgāre (meaning to tie, to bind).	From the 1570s used with the sense of to depend, to trust, typically used in the expression “to rely on something/someone".	To be consistently good and be worthy of trust
7	Auditability, Auditable	From Latin noun auditŭs, auditŭs	The sense of hearing, the act of hearing, audition. Used in the sense of official audience, judicial hearing or examination.	An “auditable" ML system should provide information on how to perform an official audience of the model. For example, this can be done by providing extra documentation and functionalities.
8	Liability, liable	From Anglo-French liable, derived from Latin lĭgo, lĭgāre (to tie, to bind)	Legal responsibility for acts.	Legal liability of a product implementing ML, particularly in the case where something goes wrong
9	Robustness, Robust	From French robuste, derived from Latin robustus, robustum.	The literal meaning is oaken, made of oak. Used in the figurative sense of strong, vigorous and resistant.	Robust ML systems are resistant, secure and reliable. Providing consistent results also in case of adversarial attacks, variations in the dataset, domain shifts, and outliers

**Fig. 3:**
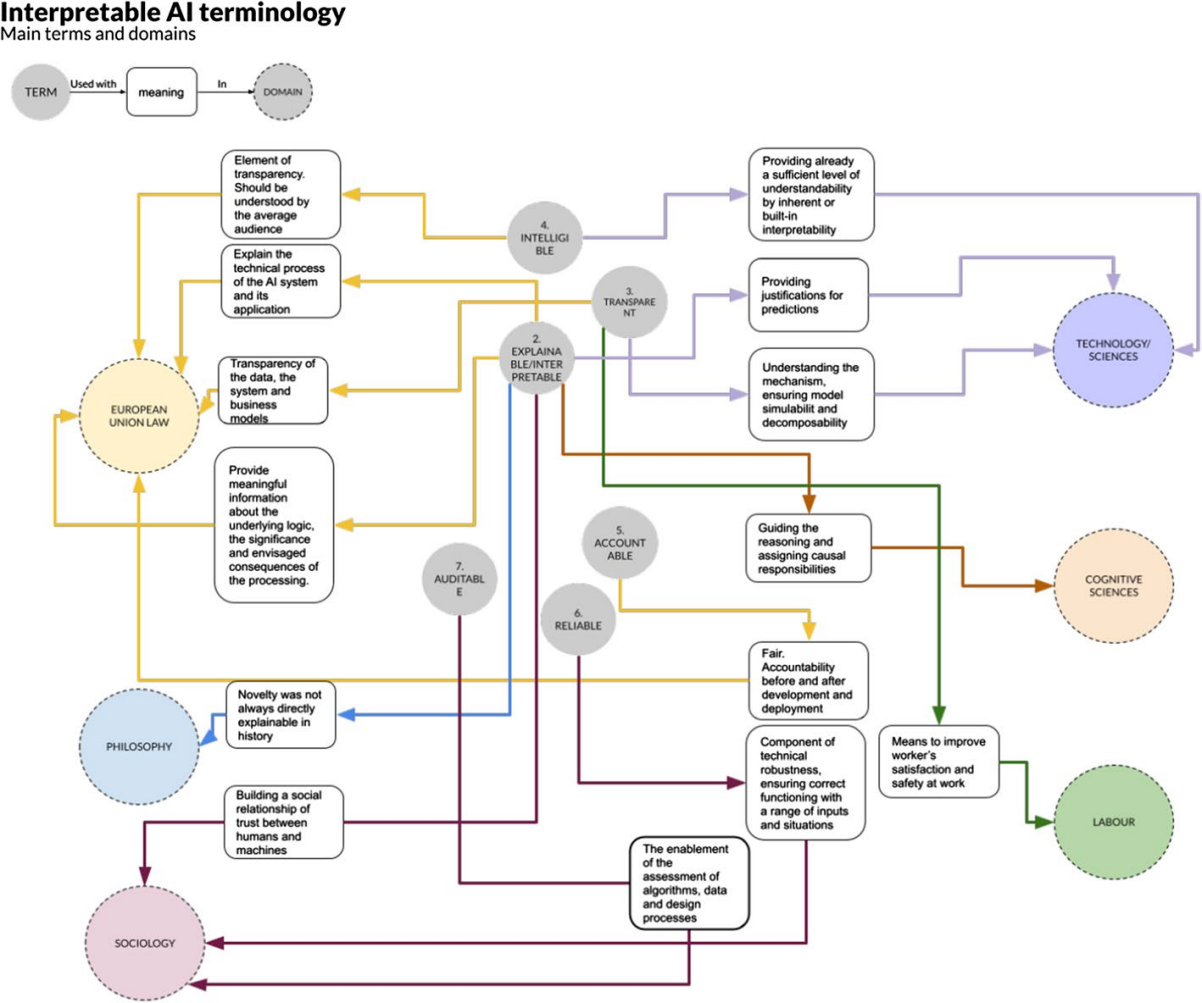
Differences of definitions in other domains than ML development. In this diagram, interpretable is equated to explainable since most of the social domains equate the two terms for simplicity

Figure 3 illustrates how some of the terms defined in Table 3 (such as intelligible, transparent, explainable, accountable, auditable and reliable) slightly change their meaning depending on the context, acquiring multiple shades and connotations as they interact with the different domains. This analysis, based on the cross-disciplinary knowledge of the people participating in the initiative, gives insights into how each domain envisions these concepts. Some conflicts in the definitions are shown as the words are used in one or another discipline. The attention towards one or more concepts is mostly heterogeneous, with some disciplines focusing more on one aspect than others. While heterogeneity in the attention to the words is legitimate and given by the intrinsic nature of each discipline, the strong changes in the meaning assigned to the same word by different disciplines may inhibit understanding and collaboration among different fields. The word *transparent* has been interpreted as “providing meaningful information about the underlying logic" in the EU legislation, whereas by technical developers this is often understood as a certain degree of understanding of the system mechanics, decomposability and simulability. In other words, if technicians and legislators were to think of the degrees of transparency of a vehicle, they would see different aspects. The former would think of pistons, fusible and the combination of these elements to the final engine. The latter would think of the degree of information available to the user about the working principles of the vehicle: starting the engine, stopping it from running, changing the direction and so on.

### A global definition of interpretable AI

As an important contribution of this work, we derive a multidisciplinary definition of interpretable AI that may be adopted in both the social and the legal sciences.

In daily language, an instance, or an object of interest, is defined as interpretable if it is possible to find its interpretation, hence if we can find its meaning (Simpson 2009). Interpretability can thus be conceived as the capability to characterize something as interpretable. A formal definition of interpretability exists in the field of mathematical logic, and it can be summarized as the possibility of interpreting, or translating, one formal theory into another while preserving the validity of each theorem in the original theory during the translation (Tarski et al. 1953. The translated theory as such assigns meaning to the original theory and it is an interpretation of it. The translation may be needed, for instance, to move into a simplified space where the original theory is easier to understand and can be presented in a different language.

From these explicit definitions, we can derive a multidisciplinary definition of interpretability that embraces both technical and social aspects: “Interpretability is the capability of assigning meaning to an instance by a translation that does not change its original validity”. The definition of interpretable AI can then be derived by clarifying what should be translated: “An AI system is interpretable if it is possible to translate its working principles and outcomes in human-understandable language without affecting the validity of the system”. This definition represents the shared goal that several technical approaches aim to obtain when applied to AI. In some cases, as we discuss in Sec. 4.4, the definition is relaxed to include approximations of the AI system that maintain its validity as much as possible. Interpretability is needed to make the output generation process of an AI system explainable and understandable to humans and it is often obtained as a translation process. Such a process may be introduced directly at the design stage as an additional task of the system. If not available by design, interpretability may be obtained by post-hoc explanations that aim at improving the understandability of how the outcome was generated. Interpretability can thus be sought through iterations and in multiple forms (e.g. graphical visualizations, natural language, or tabular data) which can be adapted to the receiver. This fosters the auditability and accountability of the system.

### A global taxonomy

In what follows we present a global taxonomy for interpretable AI, and summarize the multiple viewpoints and perspectives gathered in this work. Table 4 presents the taxonomy with further detail on domain-specific definitions used in each of the eight fields studied in this work, namely law, ethics, cognitive psychology, machine learning, symbolic AI, sociology, labour rights, and healthcare research. Brackets specify the domain in which each definition applies. If a term applies to both social and technical experts it is provided first and marked by the (global) identifier. Otherwise it is marked as the domain specific identified, i.e. EU law, sociology, etc. This table may be resorted to by practitioners in any of the above-mentioned fields to obtain a common definition for each term in the taxonomy and to inspect all the exceptions and variations of the same term in the literature. Our objective is not to impose one taxonomy above another, rather to raise awareness on the multiple definitions of each word in each domain, and to create a common terminology that researchers may refer to in order to reduce misinterpretations.

**Table 4:** Taxonomy of Interpretable AI for the social and technical sciences

Terminology	Definition in AI	Family of AI systems (technical)
Interpretability	(global) AI interpretability defines those AI systems for which it is possible to translate the working principles and outcomes in human-understandable language without affecting the validity of the system	Three families of AI systems may be identified by interpretable AI. These are (i) AI systems with built-in interpretability (ii) AI systems that are inherently interpretable (iii) AI systems that were explained by post-hoc methods. More details on these families in Table 5
	(EU law) AI interpretability defines the supply of meaningful information about the underlying logic, significance and envisaged consequences of the AI system	–
	(symbolic AI) AI interpretability includes explanations of the symbolic AI systems in symbolic language	–
	(sociology) AI interpretability must define a social relationship of trust between the human and the machine	–
Interpretability by design	(global) The translation of the system’s working principles and outcomes into human-understandable language is provided directly by the AI-system itself, interpretability being one of the tasks of the system	Two families of systems may be identified, namely (i) systems with a transparent design (e.g. introducing parameter sparsity, implementing monotonic functions Nguyen and Martínez (2019)) (ii) systems with a self-explanatory objective that generate explanations for the model predictions (e.g. interpretable decision sets Lakkaraju et al. (2016))
Post-hoc interpretability	(global) The AI system is neither inherently interpretable nor interpretable by-design, rather additional analyses are performed to generate explanations without re-training the model parameters	Six families of post-hoc interpretability methods can be identified based on the form of the generated explanations into (i) feature attribution (ii) feature visualization (iii) concept attribution (iv) surrogate explanations (v) case-based explanations and (vi) textual explanations. For further details on these categories we refer the reader to Arrieta et al. (2020) and Graziani (2021)
Local interpretability	(technical) Local interpretability is provided when interpretability analysis is performed on the system’s outcome for a single input	The family of feature attribution methods contain several approaches that provide local interpretability Ribeiro et al. (2016), Lundberg and Lee (2017), Simonyan et al. (2014), Montavon et al. (2017), Zhou et al. (2016), Selvaraju et al. (2017), Sundararajan et al. (2017), Lapuschkin et al. (2015)
Global interpretability	(technical) Global interpretability is provided when interpretability analysis is performed to explain the system behavior for a set of inputs corresponding to an entire class or multiple classes	Post-hoc interpretability methods may provide global interpretability, such as distillation techniques Frosst and Hinton (2017) and the extraction of rule lists (Chakraborty et al. (2020)
Explainability	(global) Explainable AI, also denoted as XAI, defines the branch of AI research that focuses on generating explanations for complex AI systems	The six families of post-hoc interpretability methods known as feature attribution, feature visualization, concept attribution, surrogate, case-based and textual explanations are addressed as explainable AI.
Transparency	(global) Transparency is used in AI to characterize those systems for which the role of internal components, paradigms and overall behaviour is known and can be simulated	The family of linear regression models and decision trees in low dimension are transparent and can be simulated

Brackets specify the domain in which each definition applies. Global marks a definition common to both the social and technical sciences

The following subsections explain how the proposed taxonomy adapts to the fields with their respective needs, challenges and goals in terms of ML interpretability.

### Use of the proposed terminology to classify interpretability techniques

In this section, we show how the terminology in Table 3 can be used to classify ML interpretability techniques. To do so, we group popular interpretability techniques into the families shown in Table 5. On the basis of this, Table 6 summarizes how each family of techniques can provide the properties described in Table 3. In the following, we give more insights concerning the classifications provided in Tables 5 and 6.

Due to their low complexity, models such as decision trees and sparse linear models have inherent interpretability, meaning they can be interpreted without the use of additional interpretability techniques (Molnar 2019). These methods are intelligible, according to the definition in Table 3 ID 4. Black-box models, such as deep learning models, have surpassed the performance of traditional systems over complex problems such as image classification. However, due to their high complexity, they require techniques to interpret their decisions and behavior. These techniques often involve considering a close approximation of the model behavior that may be true in the locality of an instance (i.e. local interpretability) or for the entire set of inputs (i.e. global interpretability). They can be grouped according to the following criteria: (1) scope, (2) model-agnostic, and (3) result of explanation.

The *scope* of the technique shows the granularity of the decisions that are allowed as explanation, either global or local. *Global* interpretability techniques explain the behavior of the system as a whole, answering the question “How does the model make predictions?”, while *local* interpretability techniques explain an individual or group of predictions, answering the question “How did the model make a certain prediction or a group of predictions?” (Lipton 2018).

*Model-agnostic* techniques can be applied to any model class to extract explanations, unlike model-specific techniques that are restricted to a specific model class. Interpretability techniques can also be roughly divided by their result or the type of explanation they produce, creating multiple families of techniques. It is important to note that some types of explanations are strongly preferred, as half the studies using interpretability techniques in the oncological field use either saliency maps or feature importance (Amorim et al. 2021). These techniques can produce data points that explain the behavior of the model (Kim et al. 2016; Lapuschkin et al. 2015), visualizations of internal features (Olah et al. 2017) or produce simpler models that approximate the model (Ribeiro et al. 2016; Lakkaraju et al. 2016; Lundberg and Lee 2017). It is important to choose the right technique based on its scope and family to reach the desired objective. Table 5 presents the families of techniques, their definitions and important references (Molnar 2019).

Based on Tables 1, 2 and 4 we present Table 6 where we group families of interpretability techniques based on their scope and classify them based on their suitability to achieve each of the objectives mentioned in Tables 1 and  2. To achieve interpretability as intended in Table 3 (ID 1), local techniques are preferable since they allow users to interpret the outcomes of a system and thus increase its interpretability. Global techniques can be rather inaccurate at a local level, although they are more adequate to expose the mechanisms of a system in general. The decision-making process can become more transparent (ID 3) at the local or global level, depending on the scope of the interpretability techniques. Intelligibility (ID 4) is a characteristic of inherently interpretable models. It can be achieved for more complex models by approximating the decision function either locally or globally with an inherent interpretable model. It is also important to point out that even with the model being inherently interpretable, sometimes the features being used to train the models can be hard to understand, particularly for non-experts in feature engineering.

As for accountability, systems would need to justify their outcomes and behavior to be accountable, and thus the techniques that offer any interpretability or explainability can help to achieve this. Similarly, these techniques can also be used to examine the global behavior or reasoning of local decisions and provide auditability (ID 7). Finally, Robustness (ID 9) is not achievable by only understanding the behavior of the model. It would rather require finding or producing instances that make the model misbehave, limitations of the model or data points which are outside the training data distribution.

**Table 5:** Definitions of families of interpretability techniques

Scope	Family	Definition
Inherent Interpretability	Interpretable Model	Models that are considered interpretable due to their low complexity and simple structure.
	Black-box Model	Models that are considered hard to interpret due to their high complexity and complicated structure.
Global Interpretability	Feature Visualization Nguyen et al. (2016), Olah et al. (2017)	Synthetization of new instances that help visualize features learned by the model or a specific part of the model.
	Prototype, Criticism Kim et al. (2016)	A prototype is a data instance that is representative of all the data. A criticism is a data instance that is not well represented by the set of prototypes.
	Influential Instances Koh and Liang (2017)	Data instances of which the removal has a strong effect on the trained model.
	Dependency Plot	Depicts the functional relationship between a small number of input variables and predictions.
	Global Surrogate Hinton et al. (2015)	Interpretable model that is trained to approximate the predictions of a black-box model.
	Concept Attribution Kim et al. (2018); Graziani et al. (2020)	Explain the model’s behavior based on user-friendly concepts.
	Feature Importance Lundberg and Lee (2017)	Assigns a score to input features based on how useful they are at predicting a target variable.
Local Interpretability	Local Surrogate Ribeiro et al. (2016)	Local surrogate models are interpretable models that are used to explain individual predictions of black-box models.
	Saliency Map Selvaraju et al. (2017), Lapuschkin et al. (2015)	Highlight the pixels that were relevant for a certain image prediction.
	Counterfactual Example Wachter et al. (2017)	A counterfactual explanation of a prediction describes the smallest change to the feature values that changes the prediction to a predefined output.
	Adversarial Example Goodfellow et al. (2015)	An adversarial example is an instance with small, intentional feature perturbations that cause a ML model to make a false prediction.

**Table 6:** Classification of families of interpretability techniques

Scope	Family	Interpretability	Explainability	Transparency	Intelligibility	Accountability	Auditability	Robustness
Inherent Interpretability	Interpretable Models	x	x	x	x	x	x	x
	Black-box Models	–	–	–	–	–	–	–
Global Interpretability	Feature Visualization	x	–	x	–	x	x	–
	Prototypes and Criticisms	x	–	x	–	x	x	x
	Influential Instances	x	x	x	–	x	x	x
	Dependency Plot	–	x	x	–	x	x	–
	Global Surrogate	x	x	x	x	x	x	–
	Concept Attribution	x	x	x	–	x	x	–
	Feature Importance	–	x	x	–	x	x	–
Local Interpretability	Local Surrogate	–	x	x	x	x	x	-
	Saliency Map	–	x	x	x	–	x	–
	Counterfactual Example	–	x	x	–	x	x	–
	Adversarial Example	–	–	–	–	–	x	x

At this point, we remark that interpretability techniques come with inherent risks. A desired property of interpretability is to help the end-user with creating the right mental model of an AI system. However, if one considers AI models to be lossy compression of data, then interpretability outcomes are a lossy compression of the model and are severely *underspecified*. In other words, it is possible to generate several different interpretations for the same observations. If used improperly, interpretability techniques can open new sources of risk. In some settings, interpretability outcomes can be arbitrarily changed. For example, (Aïvodji et al. 2019) demonstrate a case of “fair washing", where fair rules can be obtained that represent an underlying unfair model. It is also possible for an AI system that predicts grades to be gamed if the underlying logic is fully transparent. Model explanations can demonstrate an AI model criterion to be illegal or provide grounds for appeals (Weller 2019). Finally, transparency also conveys trade-offs involved in decisions in an explicit manner that may otherwise be hidden (Coyle and Weller 2020).

From these considerations, it follows that interpretability requires a context-based scientific evaluation. Two standard approaches for such evaluations are (a) to establish baselines based on domain insights to evaluate the quality of explanations, and (b) to leverage end-user studies to determine effectiveness. For instance, user experiments have been used for trust calibration (knowing when and when not to trust AI outputs) in joint decision-making (Zhang et al. 2020). In another interesting approach, (Lakkaraju et al. 2016) measured the teaching performance of end-users in establishing how effective explanations are in communicating model behavior with good teaching performance indicating better model understanding.

Several quantitative measures to assess explanation risks have also been proposed in the literature. A common measure using surrogates involves approximating a complex model with a simpler interpretable one. Properties of the simpler model can then help address questions on the extent of interpretability of the original model. Common measures include *fidelity*, the fraction of time the simpler model agrees with the complex one, or *complexity*, the number of elements in the simpler model a user needs to parse to understand an outcome. *Faithfulness* metrics measure the correlation between feature importance as deemed by an AI model versus deemed by an explanation. *Sensitivity* measures (Yeh et al. 2019) the degree to which explanations are impacted by non-trivial perturbations.

### Terminology in the cognitive sciences

From the point of view of the cognitive sciences, interpretability (as defined in line 1 of Table 3), is considered part of the social interaction between an AI system and a user (Hilton 1990). As the definition underlines, the concept of interpretability is strictly connected to the human ability of understanding information. The process of understanding is defined in cognitive psychology as the ability of the human brain to infer or make predictions in the semantic memory. The semantic memory is wired by connections of neurons that are created and consolidated by positive enforcement. A high-level model of such neural connections identifies areas that are specialized for reacting to specific stimuli (e.g. numbers, words, shapes, colors, actions, sounds). Depending on what kind of information is being understood, these areas may be used individually or share functions (Ward 2019). The understandability of something is thus the property of an object, may this be a model or the outcome of interpretability methods, to be understood by a human. Because the wiring of the neurons constituting the areas in the semantic memory is a result of individual experiences, understandability incorporates some degree of subjectivity and variability, e.g. what is understandable to someone may not be understandable to someone else. Users may vary greatly, so may their background and understanding of explanations. Thus to be widely applicable and useful to a variety of users, understandability shall not require any prior training of the addressees concerning the feature extraction, hyper-parameter selection and training of AI systems.

Some aspects of human explanation generation (i.e. explainability as in ID 2 Table 3) do not coincide directly with what is intuitively thought about as transparency (ID 3 in Table 3). The first difference is that explanations are selected by humans. The selection is generally biased to reflect the mental model of the explainee. Even having a complete set of causal relations, people are more likely to rely on a few causes that may explain certain key aspects of the event (Hilton 2017). It may at this point be noted that explainability should thus be intended differently from transparency, that is rather the unbiased provision of insights about the internal mechanics of an AI system.

### Social and working environment

To develop a social relationship between humans and machines, interpretability needs to act as a social contract of trust between these two parties. Trust in the system leads to reliability (as intended in ID 6 of Table 3) and this can only be built through sustained understanding. Using understanding to build trust is a well-understood social science research problem, complicated by the fact that humans accept explanations first and foremost in a highly biased manner (Lombrozo 2006). The fact that bias is part of every human understanding, however, should not limit the potential success of explainable AI. For this reason, AI explainability (ID 2 in Table 3) should be seen as a social translation, as investigated in recent studies in HCI like (Kaur et al. 2020). If only computer scientists are considered within the project ideation and development, however, there is the main risk, discussed by Miller et al. (2017), of having the helpless being led by the clueless^3^, namely having ML engineers building explainability mostly for other ML engineers. Social scientists and workers should be introduced in the analyses proposed by ML researchers, as the actual addressee and users of the algorithms. Collaborations should be built to develop types of human-computer interactions in ML that are more understandable to non-ML experts. If interpretability is not developed with the help of the social sciences, the risk of creating AI systems mainly for other researchers is high and it would undermine the efforts in building reliable and trustworthy automated systems.

AI may not be developed with the only intent of prioritizing the reduction of human input, as this may lead to the perception of AI as “inhuman” intelligence (Dick 2019). New algorithms should prioritize the creation of a relationship of trust above the desire to automate and reduce human input.

Within the realm of employment relations, work and labor markets, the concept of "democracy at work" is generating into the discussion of the criteria for AI transparency (as defined in Table 3 ID 3). Of particular importance are the employees’ rights of participation and consultation if AI algorithms are employed to make decisions at the workplace. Employees should be guaranteed the possibility to get involved in management decisions about the organization of work and of working conditions. Democracy is thus essential to let the employees create optimal conditions for work and it translates into the need of transparency if AI systems are used to manage the working personnel. In particular the workers’ autonomy (the right of a worker to intervene), skill grading and the ruling of organization and production processes should be regulated by transparent AI decisions. Transparency is thus desired to decide whether an algorithm is performing non-democratic practices, such as discrimination. It is thus intended in the sense of a means to improve the worker’s satisfaction and safety at work (see Fig. 3). Even further, it may help to identify the workplace conditions enabling discrimination in the first place.

### The EU law on interpretability

In law, there is no precise definition of AI explainability. The High-Level Expert Group on AI (AI HLEG) set up by the European Commission lists *explicability*^4^ as one of the ethical principles that must be respected in order to ensure that AI systems are developed, deployed and used in a trustworthy manner. The principle of explicability encompasses both the terms of transparency and explainability as defined in Table 3. From a legal point of view, explainability is seen as collecting meaningful insights on how a particular decision is made (Bibal et al. 2020). According to Bibal et al. (2020), it does not set the requirement for an interpretable representation of a mathematical model. Most important is that the explanation should assign meaning to the decision, i.e. so that the decision improves the explainee’s understanding^5^ of the decision generation process. It follows from the AI HLEG Guidelines that explainability should be adapted to the level of expertise and understanding of the individual concerned. Bibal et al. (2020) argue that in private decision-making, the legal requirements relate to the following four levels of ML explainability concepts: (i) providing the main features used for a decision, (ii) providing all features used for a decision, (iii) providing explanation on the way the features are combined to make the decision, and (iv) providing an understandable representation of the whole model. Wachter et al. (2017) propose the following categorization of what one may mean by an explanation of automated decision-making. Two kinds of explanations are possible, depending on whether one refers to: system functionality, i.e. the logic, significance, envisaged consequences, and general functionality of an automated decision-making system, e.g. the system’s requirements specification, decision trees, pre-defined models, criteria, and classification structures; or to specific decisions, i.e. the rationale, reasons, and individual circumstances of a specific automated decision, e.g. the weighting of features, machine-defined case-specific decision rules, information about reference or profile groups. Furthermore, one can also distinguish between an ex-ante explanation (i.e. prior to the automated decision-making taking place) and an ex-post explanation (i.e. after the automated decision has taken place) (Wachter et al. 2017). The focus of many legal scholars has been on the meaning of explainability from the data protection law point of view. The core debate has primarily focused on whether or not the General Data Protection Regulation 2016/679 (GDPR) creates a right to an explanation of an algorithmic decision, as argued by Goodman and Flaxman (2016) and further discussed by Wachter et al. (2017). The latter, in particular, argue that a non-existing “right to explanation" of a specific automated decision should not be mistaken with other GDPR provisions. The actual GDPR rather forms a “right to be informed" by claiming: (i) the right not to be subject to automated decision-making and safeguards enacted thereof (Article 22 and Recital 71); (ii) notification duties of data controllers (Articles 13-14 and Recitals 60-62); and (iii) the right to access (Article 15 and Recital 63). Others, like (Selbst and Powles 2018), point out that whether one uses the phrase “right to explanation" or not, data controllers need to provide the data subject with the “meaningful information about the logic involved, as well as the significance and the envisaged consequences of such processing for the data subject" (Article 13(2), 14(2), 15(1) of the GDPR). Such information must be meaningful to an individual confronted with a decision (Selbst and Powles 2018). The test for whether the information is meaningful should therefore be functional - explanations are a means to help a data subject act rather than merely understand the mathematical processes behind decisions (Edwards and Veale 2017). This is also in line with some of the claims done in the applicative domain at high-stakes, e.g. clinical decision-making (Tonekaboni et al. 2019).

Some scholars have studied how the legal requirements on explainability could be interpreted and applied to ML (Bibal et al. 2020). Hamon et al. (2021) used a COVID-19 use case scenario to assess the feasibility of legal requirements on algorithmic explanations. They concluded that the use of complex deep learning models in AI applications makes it hard to reconcile with the existing EU data protection law requirements, especially with regards to human legibility of explanations for non-expert data subjects. Similarly, (Edwards and Veale 2017) note that the legal concept of explanations as “meaningful information about the logic of processing" may not be provided by the kind of ML “explanations" computer scientists have developed. This further motivates the need to resort to a common ground where the objectives regarding interpretability can be discussed among the disciplines involved, for example on the basis of the taxonomy provided in this paper. It is possible that in some cases transparency or explanation rights may be overrated or even irrelevant—the problem that is often referred to as *transparency fallacy*. In many cases what the data subject wants is not an explanation-but rather for the disclosure, decision or action simply not to have occurred (Edwards and Veale 2017). In high-risk AI systems, however, the recently proposed draft Regulation on AI (the AI Act) envisions transparency as one of the obligations for the operators. Article 13 of the draft AI Act requires high-risk AI systems to be “designed and developed in such a way to ensure that their operation is sufficiently transparent to enable users to interpret the system’s output and use it appropriately." The obvious difference here, in comparison with the AI HLEG Guidelines, is that the transparency is addressed towards the users of the AI systems, that are not necessarily familiar with ML theory. This aligns with the requirement of personalized explanations discussed in Sect. 4.5 and contrasts with the current definition of transparency in the ML community where this property is rather intended as an objective peek through inside the AI algorithm.

For AI systems that interact with natural persons, e.g. an emotion recognition system or a biometric categorization system and AI systems that generate deep fakes, the draft AI Act prescribes an obligation to inform or disclose the fact that they interact or are exposed to such systems. It is interesting that even though the draft AI Act does use the very term transparency, it does not refer to the explainability and the traceability dimension that were part of the concept according to the AI HLEG Guidelines. This shows the inconsistency of the terminology from a legal point of view. One obvious solution would be to amend the text of the regulation; if not, it would be subject to interpretation by the Court of Justice of the European Union that is likely to rely on other branches of science to complement the legal gaps, which shows the clear necessity of unified taxonomy.

### An ethical point of view

The requirement of interpretability is often made on the basis of an analogy with human decision-making (Coeckelbergh 2020). We expect bankers to explain why they reject a loan, physicians to explain why they discontinue treatment and politicians to explain why they want to implement a certain policy. This requirement is often based on the idea of transparency: that seeing how a phenomenon happens generates accountability and the possibility of change (Ananny and Crawford 2018). The interpretation of phenomena in this sense derives from the epistemological concerns being debated since antiquity in philosophy. In the historical sense (in Table 3), interpreting has to do with understanding a particular course of action or decision-making and ethical concerns have to do with providing reasons for moral choices. Even prior to that, interpretation has been primarily a religious issue, namely concerning the interpretation of the holy scripture, which was supposed to transmit the word of God, in a way such that the true meaning of the text would be preserved.

Unlike other technologies, interpretation is one of the primary ethical concerns that are raised with the application of AI. While other technologies are also able to replace human functions (e.g., a walking stick takes over the function of a leg), AI is arguably the first technology that has the capacity to make decisions. And this raises both the epistemological question of *why* certain decisions were made by an AI system, as well as the ethical question of whether *good reasons* can be given for this decision, in case it is of ethical significance.

What sets the ethical discussion apart from the technical perspective in Sect. 4.4, is its primary focus on the ethical value of an explanation, rather than in its epistemic value (Robbins 2019). That is, a causal chain leading to the damage needs to be provided if an AI-generated decision may affect a human being.

As scholars have argued, however, human beings often do not need complete causal chains of explanation (Coeckelbergh 2020). This opens up some new ethical issues and problems such as the intentional concealing of information, which may be obtained even by simply providing explanations of which the understandability is limited by the requirement of prior expert knowledge (Ananny and Crawford 2018). A patient might not be helped by a full causal explanation of a diagnosis but rather by a trustworthy account of understandable reasons expressed in clear and simple language.

From this perspective, we may raise three overarching ethical concerns of interpretable AI. First, there is the concern of “sacrifice". Because interpretation is always situated between the system and the user, it generates the inevitable risk of omission during interpretation. This can be due to either oversimplification (simplifying the model dynamics missing out on important technical details) or to overcomplexify (providing too technical explanations most users cannot grasp) (Nissenbaum 2011). Interpretation therefore inevitably sacrifices meaning. Second, we should be concerned about “hospitality", here intended as a common ground of understanding between strangers that aims to remedy the potential of conflict. Interpretation requires building bridges between different world visions, for instance between a physician and a patient, or a civil servant and a citizen. Third, interpretation raises the question of professional virtues. It is often part of a particular profession (a notary, a physician, a school teacher) to uphold certain standards of excellence in providing interpretability, for instance under the heading of the virtue of “fidelity". Importantly, what these standards mean in practice can differ significantly between different professional contexts.

In light of the above three (and other) ethical challenges, researchers have to consider how the ethical interpretability of AI systems should be realized in practice. Often, this requires finding ways in which humans and AI systems are able to work together in providing interpretations that are related to practices, sensitive to context, and provide good reasons for making ethical choices if required.

### Not only humans: XAI in intelligent autonomous systems

Virtual agents are the most common embodiment of symbolic AI (Russell and Norvig 2002). They can operate singularly, in a cooperative or adversarial fashion (within Multi-Agent Systems—MAS). The agents composing intelligent autonomous systems (MAS) are hardware/software-based computer systems characterized by any or all of the following: (i) autonomy (no direct intervention or human control), (ii) social ability (free to interact with other agents and humans), (iii) reactivity (perception of their environment and according reactions), and (iv) pro-activeness (being goal-directed, they can take the initiative) (Franklin and Graesser 1996). MAS have increasingly become part of modern society and as such are incorporated in an increasing number of everyday tasks (Calvaresi et al. 2017).

Beyond their symbolic nature, modern agents can also leverage sub-symbolic algorithms (i.e., ML and DL), integrating them into their reasoning processes (Schwartz 2014). While symbolic agents are explainable by design (being mainly rule-based), the behavior of sub-symbolic or hybrid agents can result in being opaque for both human users and other agents. Such opacity harms the reputation of the single agents and the trust into the overall intelligent system (Anjomshoae et al. 2019; Ciatto et al. 2020). In the last decades, the majority of the articles in explainable agents focused on making intelligent systems understandable primarily to humans (Rosenfeld and Richardson 2019; Anjomshoae et al. 2019; Guidotti et al. 2018). Bridging symbolic and sub-symbolic approaches is called neuro-symbolic integration (Stammer et al. 2021; Sarker et al. 2021). For example, (De Raedt et al. 2019) proposed to adopt neuro-symbolic and probabilistic approaches, (Riveret et al. 2015) to adopt neuro-argumentative techniques, and (Besold and Kühnberger 2015) proposed two paths to achieve such an integration. Nevertheless, current research indicates that the forthcoming decades will focus on the full development of conversational informatics (Nishida 2014; Calvaresi et al. 2021). MAS are modeled after human societies and within MAS agents communicate with each other, sharing syntax and ontology. They interact via the Agent Communication Languages (ACL) standard [?] shaped around Searle’s theory of human communication based on speech acts (Searle et al. 1969). Therefore, multi-agent interpretability and explainability require multi-disciplinary efforts to capture all the diverse dimensions and nuances of human conversational acts, transposing such skills to conversational agents (Ciatto et al. 2019, 2020). Equipping virtual entities with explanation capabilities (either directed to humans or other virtual agents) fits into the view of socio-technical systems, where both humans and artificial components play the role of system components (Whitworth 2006). Ongoing international projects revolve around these concepts. For example, they are tackling intra- and inter-agent explainability (EXPECTATION), actualizing explainable assistive robots (COHERENT), countering information manipulation with knowledge graphs and semantics (CIMPLE), and relating action to effect via causal models of the environment (CausalXRL) 6. Explainable agents can leverage symbolic AI techniques to provide a rational and shareable representation of their own specific cognitive processes and results. Being able to manipulate such a representation allows building one or more personalized explanations to meet the explainee (human and virtual) background and boost the success of the explanation process and overall interaction.

## A case study: the medical domain

In this Section, we present a case study in a medical scenario. We show how each of the perspectives from the multiple domains (i.e. from the legislation, cognitive, social, ethical, philosophical, rights at work, ML and symbolic AI) comes into play in a possible use case. As argued by Tonekaboni et al. (2019) and Banja et al. (2022), the application of ML to clinical settings represents a relevant use case for interpretability, motivated by the high stakes, the complexity of the modeling task and the need for reliability. From the legal perspective, clinicians are the sole people legally accountable for any diagnosis and decision-making, hence accepting ML suggestions is seen as taking an acknowledged risk that may affect the survival and life quality of the patient. As the cognitive sciences suggest, clinicians should be able to revise their mental model of the AI system to be able to understand the principles applied by the systems’ decision-making, ensuring the reliability of the systems. It is only through time and sustained use that a social relationship of trust between the physician and the automated system can be installed. Interpretability is to be sought in the medical application not only for the sake of the philosophical and epistemic value of explanations per se, but also as an ethical requirement to provide a factual, direct and clear explanation of the decision-making process, especially in the event of unwanted consequences" (Floridi et al. 2018; Robbins 2019). An AI-generated decision arguably needs to be interpretable if it can affect a human being. Given the high cost of making a mistake, the ML application cannot be allowed to take decisions independently, differently from other contexts where ML tools are used lightly, e.g. recommendation systems. This sets a major requirement to ensure the well-being of the physicians in the workplace, making sure that their confidence with the tools may increase over time and provide them with sufficient transparency to take the decisions on whether to rely or not on the AI system. To satisfy the requirements set by this analysis from the social sciences, the ML and symbolic-AI tools deployed for clinical use should interact with the experts for which technical solutions must be developed.

The interaction between humans and ML systems is a non-trivial task. Human reasoning is mostly based on high-level concepts that interact with each other to form a semantic representation. These interactions with semantic meaning are not necessarily represented by ML models that mostly operate on numeric features such as input pixel values, internal activations and model weights (Kim et al. 2018). When the features used by the model are expressed in clinical terms, the interaction of the clinicians with the system is enhanced and can lead to successful cooperation. An example is the case described in Caruana et al. (2015). Despite its high performance, the model for pneumonia risk detection had a hidden flaw. Cases of pneumonia with concurring asthma were assigned a lower risk of death than those without, despite the presence of this condition being known to worsen the severity of the cases. A correct prediction would have been the opposite diagnosis given the high risk of death. The misleading correlation (i.e. presence of asthma thus low risk of death from pneumonia) was rather a consequence of the effective care given to these patients by healthcare specialists that were promptly reacting to reduce the risk of death, and as a consequence lowering the recorded risk for these patients. The misleading feature “presence of asthma" was captured by the interpretability analysis and it was promptly understood by physicians since it was expressed as a clinical feature.

It is now worth pointing out that, as described by Asan et al., “maximizing the user’s trust does not necessarily yield the best decisions from a human-AI collaboration" and that the optimal trust level can be achieved when the user knows when the model makes errors. After recalling that the role of humans in the practical applications of AI has been overlooked (Asan et al. 2020; Verma et al. 2021), they suggest that achieving such an understanding of both strengths and weaknesses of the models requires a combination of three main elements: (i) increasing transparency, (ii) ensuring robustness (Briganti and Le Moine 2020) and (iii) encouraging fairness. Concerning (i), XAI was mentioned as the most promising approach to alleviate the black-box effects (Morin et al. 2018; Reyes et al. 2020; Verma et al. 2021). In addition, we believe that current AI model lifecycles are often too short for the user to acquire a sufficiently high confidence, where novel approaches, or even retrained versions of the same algorithm are constantly released, sometimes with only little quantitative performance improvement. This can be compared to a situation where drivers must flawlessly master their vehicle while the latter is continuously changing shape and characteristics. One must therefore foster patience to achieve an adequate level of trust, which involves an intimate relationship between the end-user and a particular instance of the model to seize the situations where the model is working well and where it does not. This was *de facto* encouraged by the U.S. Food and Drug Administration (FDA), which as of June 2021 only approved static algorithms. However, as pointed out by Pianykh et al. the performance of static AI algorithms tends to degrade over time, owing to the naturally occurring changes in local data and the environment (Pianykh et al. 2020). Furthermore, the access to a large collection of well-curated, expert-labeled data from a source that has high relevance to the studied population and the question asked is also a severe barrier for widespread adoption in the clinics (Willemink et al. 2020). We can conclude that an optimal model lifecycle has yet to be discovered to balance between model performance and robustness as well as adequate user trust and data access to optimally train AI models.

## Conclusion

This work proposes an in-depth discussion of the terminology in interpretable AI, highlighting the risks of misunderstanding that exist if differing definitions are employed in the technical and social sciences. As noted by the experts, there are important gaps between how, for example, the legal legislation shows the notion of transparency and the meaning that is assigned to this word by ML experts and developers. While in the first case transparency is intended as a subjective property that is influenced by the receiver’s understanding and prior knowledge, in the technical sciences transparency is rather seen as an objective property that is not influenced by the receiver of the information. Similarly, the notion of interpretability is seen as the creation of a social contract of trust by social sciences, whereas this is yet too often intended as the explanation of the automated generation process of the AI system by most AI experts.

The taxonomy proposed in this paper has the objective to harmonize the terminology used by lawyers, philosophers, developers, physicians and sociologists, with the goal of building a solid basis for discussing the future of AI development in a multidisciplinary setting. We show how the proposed terminology is used in multiple domains and also its versatility to social and technical discussions. By discussing these points on the concrete application of the medical domain we show that the need for a common terminology is real and that further reflection is needed to define how effective human-machine cooperation can be established. Without the help of the social sciences, it would not be possible to obtain a sustainable human-machine partnership and further research needs to be pursued at the frontier of the social and technical sciences. This paper may then constitute a strong foundation for scientists and humanists to collaborate and interact on such matters.

## Footnotes

1 https://taxonomyinterpretableai.wordpress.com/, as of October 2021.

2 https://www.youtube.com/watch?v=aVLCDORsqmo, as of February 2022.

3 In the original paper, this problem is formulated as that of “the inmates running the asylum".

4 https://www.europarl.europa.eu/cmsdata/196377/AI%20HLEG_Ethics%20Guidelines%20for%20Trustworthy%20AI.pdf, as of February 2022.

5 Intended in the scientific sense used in cognitive psychology (see Sect. 4.5).

6 Projects within the CHIST-ERA pathfinder programme for research on future and emerging information and communication technologies https://www.chistera.eu/projects.
